# Resveratrol did not alter blood pressure in rats with nitric oxide synthase-inhibited hypertension

**DOI:** 10.5830/CVJA-2016-069

**Published:** 2017

**Authors:** Mehmet Aydin, Ibrahim Susam, Ali Kemal Cabuk, Buket Gungor, A Secil Akdur, Hakki Engin Aksulu, Coskun Silan, Coskun Silan, Gizem Cabuk

**Affiliations:** Department of Cardiology, Tepecik Training and Research Hospital, Izmir, Turkey; Department of Cardiology, Tepecik Training and Research Hospital, Izmir, Turkey; Department of Cardiology, Tepecik Training and Research Hospital, Izmir, Turkey; Department of Clinical Trials, Turkish Medicines and Medical Devices Agency, Turkish Ministry of Health, Ankara, Turkey; Department of Clinical Pharmacology, Canakkale State Hospital, Turkish Ministry of Health, Canakkale, Turkey; Department of Pharmacology, School of Medicine, Canakkale Onsekiz Mart University, Terzioglu Campus, Canakkale, Turkey; Department of Pharmacology, School of Medicine, Canakkale Onsekiz Mart University, Terzioglu Campus, Canakkale, Turkey; Nanoscience and Technology Research and Application Center (NANORA C), Canakkale Onsekiz Mart University, Terzioglu Campus, Canakkale, Turkey; Department of Cardiology, Buca Seyfi Demirsoy State Hospital, Izmir, Turkey

**Keywords:** hypertension, NOS, resveratrol, anti-oxidant, sodium excretion

## Abstract

**Background:**

Inhibition of nitric oxide synthase (NOS) is a well-known experimental model of hypertension (HT). It was shown that oxidative stress contributes to the pathogenesis of HT. Resveratrol is a potent anti-oxidant that is found in red grapes, peanuts and red wine. It improves the NO response and increases endothelial NOS expression, which causes endothelium-dependent vasorelaxation as well as renal vasodilation. We aimed to explore the effects of resveratrol on blood pressure, the water–salt balance and sodium excretion as a reflection of renal function in NOS-inhibited rat models.

**Methods:**

Thirty-five male Sprague-Dawley rats (200–250g) were used in this study. In order to obtain hypertension models, an NOS inhibitor, N-nitro-L-arginin (L-NNA) was used. The rats were randomly divided into five groups: controls (given water and 0.8% salty diet) and four groups [given L-NNA, resveratrol (RSV) eluent, RSV, and L-NNA + RSV]. Blood pressures were measured indirectly by the tailcuff method on the first, seventh and 10th days. At the end of the study protocol (10th day), fluid balance, glomerular filtration rate, fractional sodium excretion, and blood and urinesodium and creatinine levels were measured.

**Results:**

At the end of the study protocol, blood pressures were higher in only the L-NNA group (117.8 ± 3.5 vs 149.5 ± 2.1 mmHg; p < 0.05), as expected. Additional applications of RSV with L-NNA could not prevent the increase in blood pressure (122.8 ± 7.3 vs 155.4 ± 4.4 mmHg; p < 0.05). There were no remarkable changes in water–salt balance and renal function with the application of resveratrol.

**Conclusion:**

Resveratrol was unable to prevent or reverse blood pressure increase in NOS-inhibited rats.

## Background

Essential hypertension (HT) is one of the leading causes of preventable deaths and a major risk factor for serious disorders such as coronary heart disease, heart failure, peripheral vascular disease, renal failure and stroke. Pathogenesis of HT is multifactorial and synthesis and/or release of nitric oxide (NO), which regulates local blood flow and modulates sodium reabsorption, plays a role in this process.

In order to shed light on the multifactorial pathophysiological mechanisms of hypertension and to improve preventative and therapeutic strategies, many experimental models have been used. One of these experimental models is impairment of NO production in the blood vessel layer, which is a major pathway for the development of hypertension, by using nitric oxide synthase (NOS) inhibitors.^1^,^2^

Acute or chronic inhibition of NO production by NOS inhibitors causes hypertension,^3^-^7^ and the degree of elevation of blood pressure is dose and time dependent. With total inhibition of NOS with high doses, increased periferal resistance comes to the fore as the underlying cause; however, water and salt retention, activation of the symphathetic system and oxidative stress are important contributors.^8^-^10^

Oxidative stress was shown to be related to inadequate natriuresis and vasodilatation by means of impaired expression or function of renal dopaminergic receptors, however the mechanism is not clear.^11^ The exact role of oxidative stress in the development of HT via NOS inhibition and the regulatory effect of the anti-oxidant system in this process remains unresolved.

Resveratrol (3,4′,5-trihydroxy-trans-stilbene) (RSV) is a type of natural phenol found in red grapes, peanuts, red wine and other polyphenol-rich food. Anti-proliferative,^3^,^4^ anti-inflammatory,^5^ anti-oxidant^5^-^7^,^12^-^14^ and cardioprotective^15^ effects of resveratrol have been shown in different experimental models so far.

Human studies established that acute administration of RSV generated dose-dependent improvement of endotheliumdependent vasodilatation.^16^ Aminopiridin-sensitive potassium channels play a role in that process and a potassium-independent pathway (propably related to voltage-dependent calcium channels) is also thought to be responsible for the vasodilatory efects of RSV.^17^,^18^ Furthermore, it was shown that aortic vasodilation, with a low dose of RSV, was generated via the endothelial NOS effect.^19^

In our study, we aimed to investigate the effect of resveratrol on blood pressure in rats that become hypertensive via NOS inhibition with the application of L-NNA in doses that cause mild hypertension.^20^ Changes in parameters related to water–salt balance and renal functions were also analysed.

## Methods

Male Sprague-Dawley rats (230–260 g) from Harlan were housed under standard conditions with a 12-hour light–dark cycle in standard cages in a room with a controlled humidity of 40% and a temperature of 22°C. They had ad libitum access to food and water for 10 days.

Experimental procedures were in agreement with institutional and legislator regulations and approved by the local ethics committee for animal experimentation.

The rats (n = 35) were randomly divided into five groups (n = 7 in each group): control [intraperitoneal (i.p.) 1 ml 0.9% serum physiological solution applied for 10 days], L-NNA (15 mg/100 ml L-NNA given with drinking water for 10 days), RSV-E [1 ml resveratrol eluent (20% ethanol) i.p. applied for 10 days], RSV50 (50 mg/kg resveratrol i.p. applied for 10 days) and L-NNA + RSV50 (15 mg/100 ml L-NNA given with drinking water and 50 mg/kg resveratrol i.p. applied for 10 days).

The amount of consumed water was quantified every day and all applications were performed at the same time of day. The dose of L-NNA was calculated from the amount of consumed water and the drinking water of all groups was refreshed every day.

Each subject was placed in a separate box in a quiet area. Atail-cuff pletysmograph (MAY BPHR 9610-PC TAIL-CUFF Indirect Blood Pressure Recorder, Ankara, Turkey) and its sensor were fixed to their tails, which were warmed up to 37–38°C for 10–20 minutes, until it picked up regular signals and obtained pulses. Systolic blood pressure and heart rate were measured with the indirect tail-cuff method on the first, seventh and 10th days of the study by investigators who were blinded to the study protocol. An average of three measurements was recorded on each occasion.

All rats were put into metabolic cages at the end of study protocol. The total water intake and urine output were determined for 24 hours. We added 0.1 ml 6N HCl to the urine containers and kept the samples in the dark. Urine samples were put into Eppendorf tubes and stored at –80°C (Sanyo Ultra Low Temperature Freezer MDF-U4086S).

At the end of the experiment, the animals were anesthetised with 20% urethane (1 g/kg, i.p.). Blood samples were collected by heart puncture, and serum samples were obtained after centrifugation of the blood at 5 400 rpm for 10 minutes and stored at –80°C. We measured urea, creatinine and sodium levels in the blood and urine samples with a Roche Cobas 6000 autoanalyser (Mannheim, Germany).

 Fluid balance, sodium clearance rate (CNa), glomerular filtration rate (GFR) and fractional sodium excretion (%FENa) were calculated using the following formulae:

Fluid balance = water intake – urine volume

C_Na_ = (urine sodium x UFR) / plasma sodium

GFR = (urine creatinine x plasma creatinine) / UFR

%FE_Na_ (plasma creatinine x urine sodium) / (plasma sodium x urine creatinine) x 100

## Statistical analysis

All statistical analyses were performed with IBM SPSS Statistics 16 software (SPSS Inc, Chicago, IL, USA). Data are expressed as mean ± standard error. Blood pressure values were compared with the Student’s t-test and biochemical values via one-way analysis of varience (ANOVA) with post hoc Bonferroni comparison. All p-values were two-tailed and p < 0.05 was considered to be statistically significant.

## Results

**Fig. 1.**
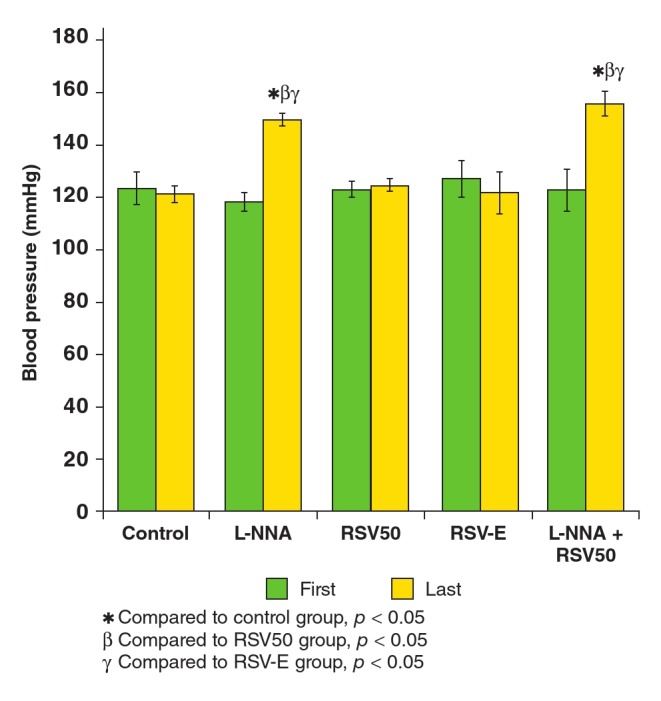
The first and the last measured blood pressures of the study groups.

The body weight gains of all groups were similar and are shown in Table 1. The first measured blood pressure values (before the protocol) were similar between the groups (Table 2, Fig. 1). At the end of the study protocol, blood pressures were higher in the L-NNA (117.8 ± 3.5 vs 149.5 ± 2.1 mmHg; p < 0.05) and L-NNA + RSV50 (122.8 ± 7.3 vs 155.4 ± 4.4 mmHg; p < 0.05) groups (Table 2, Fig. 1).

**Table 1:** Weight gain in the study groups

*Groups (n = 7)*	*First day (g)*	*Last day (g)*
Control	154 ± 5.5	199.4 ± 7.2
L-NNA	155 ± 3.3	209.0 ± 3.3
RSV50	151 ± 2.8	188.5 ± 4.2
RSV-E	188 ± 5.6	214.8 ± 16.9
L-NNA + RSV50	186 ± 6.3	194.3 ± 5.2

**Table 2:** Blood pressure measurements of the study groups at the beginning and end of the study

*Groups (n = 7)*	*First measured (mmHg)*	*Last measured (mmHg)*
Control	123.1 ± 5.5	121.1 ± 3.5
L-NNA	117.8 ± 3.5	149.5 ± 2.1*^βγ^
RSV50	122.4 ± 3.8	124.2 ± 2.4
RSV-E	126.6 ± 6.4	121.7 ± 7.8
L-NNA + RSV50	122.8 ± 7.3	155.4 ± 4.4*^βγ^

*Compared to the control group, p < 0.05; βcompared to the RSV50 group, p < 0.05; γcompared to the RSV-E group, p < 0.05.

L-NNA (42.0 ± 1.0) and RSV (44.7 ± 3.6 ml) application did not alter the water intake, whereas it was found to be significantly lower in the L-NNA + RSV50 (25.9 ± 2.9 ml) and RSV-E (26.8 ± 4.3 ml) groups compared to the control group (33.7 ± 4.1 ml) (Table 3).

**Table 3:** Water intake, urine volume and water balance in the study groups

*Groups (n = 7)*	*Water intake (ml)*	*Urine volume (ml)*	*Water balance (ml)*
Control	33.7 ± 4.1	11.6 ± 1.2	2 2.1 ± 4.1
L-NNA	42 ± 1.0	14 ± 0.5	28.0 ± 1.0
RSV50	44.7 ± 3.6	10.1 ± 0.3^αγ^	34.6 ± 3.5*
RSV-E	26.8 ± 4.3^αβ^	3.3 ± 1.1*^αβ^	23.5 ± 3.7
L-NNA + RSV50	25.9 ± 2.9^αβ^	12.9 ± 1.2*	13 ± 2.4^αβ^

*Compared to the control group, p < 0.05; αcompared to the L-NNA group, p < 0.05; βcompared to the RSV50 group, p < 0.05; γcompared to the RSV-E group, p < 0.05.

Compared to the control group (11.6 ± 1.2 ml), urine volume did not change in the L-NNA (14.0 ± 0.5 ml), RSV50 (10.1 ± 0.3 ml) and L-NNA + RSV50 (12.9 ± 1.2 ml) groups. However, interestingly, urine volume (3.3 ± 1.1 ml) as well as water intake was significantly lower in the RSV-E group (Table 3).

**Fig. 2.**
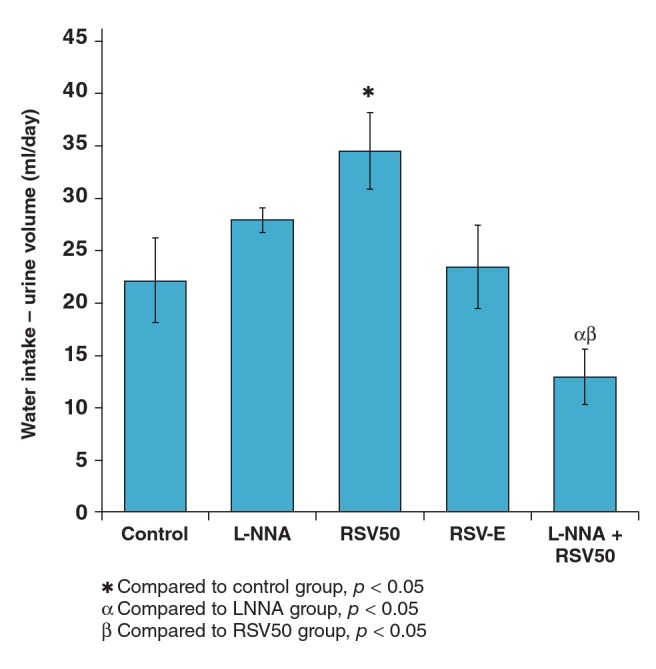
Fluid balances of the study groups.

Although the application of RSV with L-NNA decreased the fluid balance (13.0 ± 2.4 ml), RSV application alone increased it (34.6 ± 3.5 ml). L-NNA application alone did not alter (28.0 ± 1.0 ml) the fluid balance compared to the control group (22.0 ± 4.0 ml) (Table 3, Fig. 2).

Serum and urine sodium concentrations (Table 4) and urea and creatinine (Table 5) levels were similiar between the groups. Applications did not alter the values measured at the end of the study compared with those measured at the beginning of the protocol (data not shown).

**Table 4:** Serum sodium concentrations and 24-hour urine samples

*Groups (n = 7)*	*Serum Na (mEq/l)*	*24-hour urine Na (mEq/l)*
Control	144.0 ± 0.53	0.84 ± 0.11
L-NNA	143.4 ± 0.4	0.72 ± 0.07
RSV50	143.7 ± 0.5	0.86 ± 0.14
RSV-E	143.5 ± 0.4	0.84 ± 0.15
L-NNA + RSV50	143.2 ± 0.4	0.89 ± 0.16

**Table 5:** Creatinine and urea levels in serum and 24-hour urine samples

*Groups (n = 7)*	*Serum creatinine (mg/dl)*	*Serum urea (mg/dl)*	*24-hour urine creatinine (mg/dl)*	*Control*
Control	0.38 ± 0.01	44.60 ± 2.63	57.03 ± 2.81	0.58 ± 0.03
L-NNA	0.36 ± 0.02	48.67 ± 2.74	52.60 ± 4.15	0.47 ± 0.05
RSV50	0.39 ± 0.03	40.31 ± 2.96	52.21 ± 2.86	0.48 ± 0.04
RSV-E	0.37 ± 0.02	43.65 ± 3.77	53.58 ± 3.38	0.55 ± 0.03
L-NNA + RSV50	0.38 ± 0.01	44.09 ± 0.73	58.44 ± 3.60	0.56 ± 0.01

**Fig. 3.**
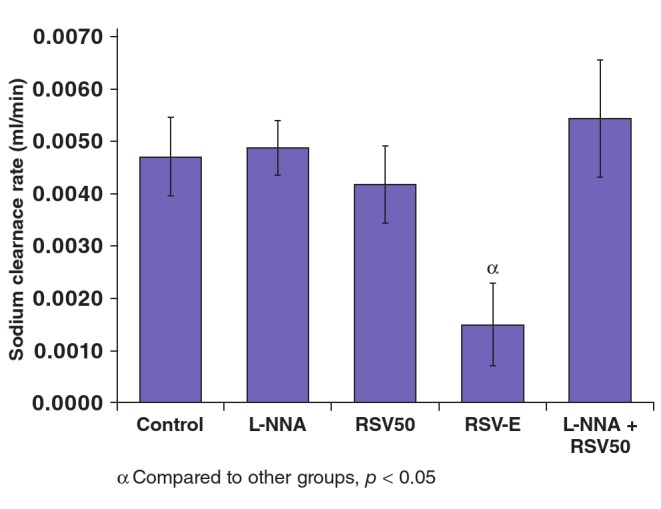
Sodium clearance values of the study groups.

RSV and L-NNA application alone did not alter CNa, however it was lower in the RSV-E (0.0015 ± 0.0007 ml/min) group compared to both the RSV50 (0.0042 ± 0.0007 ml/min) and L-NNA (0.0055 ± 0.0011 ml/min) groups (Table 6, Fig. 3).

**Table 6:** Sodium clearance rate (CNa), glomerular filtration rate (GFR) and fractional sodium excretion (%FENa) values

*Groups (n = 7)*	*C_Na_ (ml/min)*	*GFR (ml/min)*	*%FE_Na_*
Control	0.0047 ± 0.0007^γ^	1.25 ± 0.19^γ^	0.38 ± 0.04
L-NNA	0.0049 ± 0.0005^γ^	1.30 ± 0.16^γβ^	0.42 ± 0.07
RSV50	0.0042 ± 0.0007^γ^	0.87 ± 0.08^γ^	0.53 ± 0.11
RSV-E	0.0015 ± 0.0007	0.32 ± 0.09^*β^	0.41 ± 0.09
L-NNA + RSV50	0.0055 ± 0.001^γ^	1.33 ± 0.14^γβ^	0.42 ± 0.08

*Compared to the control group, p < 0.05; αcompared to the L-NNA group, p < 0.05; βcompared to the RSV50 group, p < 0.05; γcompared to the RSV-E group, p < 0.05.

**Fig. 4.**
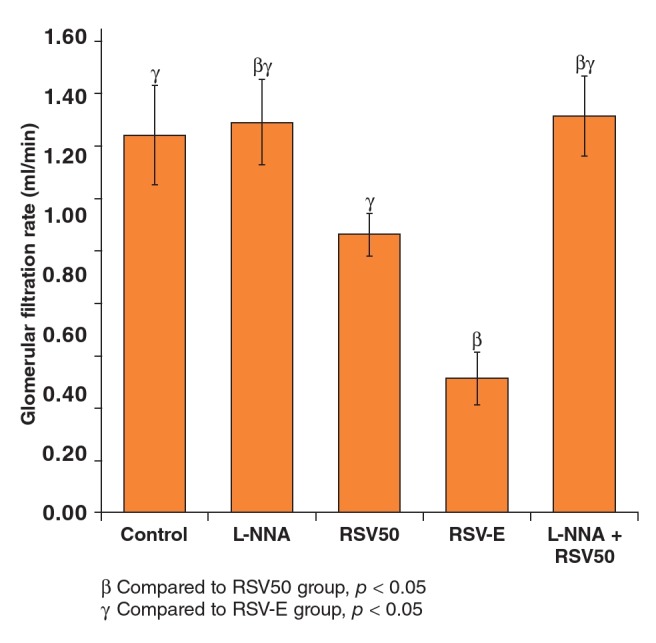
Glomerular filtration rates of the study groups.

GFR was significantly lower in the RSV-E group (0.32 ± 0.09 ml/min) compared to the other groups and it was also lower in the RSV50 group (0.87 ± 0.08 ml/min) compared to the L-NNA (1.30 ± 0.16 ml/min) and the L-NNA + RSV50 (1.33 ± 0.14 ml/min) groups (Table 6, Fig. 4).

**Fig. 5.**
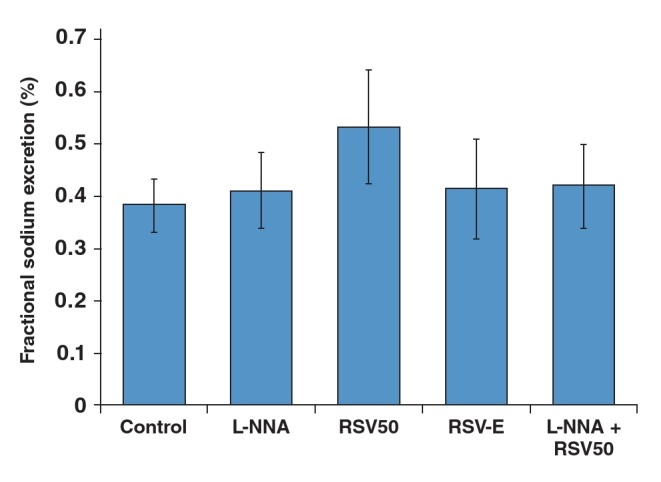
Fractional sodium excretion values of the study groups.

Fractional sodium excretion values were similar in all groups (Table 6, Fig. 5).

## Discussion

It has been demonstrated that vascular oxidative stress plays an important role in the pathogenesis of essential hypertension,^21^ and many experimental studies have been published using anti-oxidants to prevent the development of hypertension or to decrease blood pressure. Hu et al.^22^ showed that apocynin [a nicotinamide dinucleotide phosphate (NADPH) oxidase inhibitor] application prevented and reversed dexamethasoneinduced (via NADPH oxidase-mediated superoxide production) hypertension in rats. Another study revealed that tempol [a superoxide dismutase (SOD) mimetic] application decreased blood pressure and renal vascular resistance in spontaneously hypertensive rats by eliminating unfavourable peroxynitrite formation from the superoxide by competition with NO.^23^

We aimed to investigate the effects of RSV in preventing the development of hypertension, which was induced by NOS inhibition. RSV application alone did not alter blood pressure in the normotensive rats. Application of RSV plus L-NNA did not reverse the blood pressure increase induced by L-NNA. This may have been related to the dose of RSV or the length of the application period. Various protocols have been used in other studies.

Bhatt et al.^24^ gave RSV dissolved in drinking water to rats at a concentration of 50 mg/l for 10 weeks and revealed that the development of hypertension was attenuated in the spontaneously hypertensive rats. Gordish et al.^25^ administered RSV at a dose of 5 mg/kg to rats via the femoral vein and proved that the acute renal vasodilatory effect of RSV was mediated by increased NO production/bioavailability and its superoxidescavenging effect. We did not measure oxidative stress markers such as malondialdehyde (MDA), however we possibly achieved sufficient antioxidant effect by the RSV aplications.

In addition, it was shown that abnormalities in vascular NO production and transport resulted in hypertension due to endothelial dysfunction. RSV increased NO synthesis and functioned as a potent in vivo anti-oxidant.^26^

Interestingly, in the RSV50 group, the water balance was significantly higher compared to the control group despite no significant changes in water intake and urine output. Application of L-NNA plus RSV decreased the water balance compared to the application of RSV only, however it was not significantly different compared to the control group. Decreased urine volume may have been related to diminished water intake, as found in previous studies.^27^-^29^

C_Na_ and GFR values were lower in the RSV-E group and these findings were attributed to the RSV eluent (20% ethanol), which is comparable with the study of Barrero and co-workers.^30^ They showed that creatinine and sodium clearance decreased after ethanol application in rats.

The kidneys play a critical role in long-term control of blood pressure. Reduction in renal sodium excretion or a rightward shift in the pressure–natriuresis relationship results in persistent hypertension, and NO plays an important role in this process by regulating the renal response to changes in perfusion pressures. Suprisingly, renal functional parameters were not affected by NOS inhibition in our study.

Tolins et al.^31^ revealed that renal and systemic vascular resistance increased, and renal blood flow and sodium excretion were decreased by NOS inhibition. Griffin et al.^32^ showed that Sprague-Dawley rats from Harlan, which exhibited the expected hypertension, proteinuria and glomerular damage, and those from Charles River, which showed a blunted increase in blood pressure and a resistance to nephropathy, exhibited largedifferences in susceptibility to nephropathy by L-NAME-induced NOS inhibition over a period of four weeks. We used L-NNA instead of L-NAME for NOS inhibition in order to obtain earlier blood pressure increase, and we evaluated fluid balance, CNa, GFR and %FENa but not proteinuria and morphological parameters of renal damage over a period of 10 days.

## Conclusion

Although it is a potent anti-oxidant and inceases NO production/bioavailibilty, resveratrol was incapable of preventing the development of hypertension or reversing the blood pressure increase in L-NNA-induced hypertension models in our study. We cannot generalise this finding, as resveratrol is not a good candidate for the treatment of hypertension developed via the NOS-inhibition pathway. We suggest that further studies are needed to assess this hypothesis, with higher doses and/or longer periods of time.

## References

[1] Hill C, Lateef AM, Engels K, Samsell L, Baylis C (1997). Basal and stimulated nitric oxide in control of kidney function in the aging rat.. Am J Physiol.

[2] Linder L, Kiowski W, Buhler FR, Luscher TF (1990). Indirect evidence for release of endothelium-derived relaxing factor in human forearm circulation in vivo. Blunted response in essential hypertension.. Circulation.

[3] Savio M, Coppa T, Bianchi L, Vannini V, Maga G, Forti L (2009). The resveratrol analogue 4,4’-dihydroxy-trans-stilbene inhibits cell proliferation with higher efficiency but different mechanism from resveratrol.. J Biochem Cell Biol.

[4] Hsieh TC (2009). Antiproliferative effects of resveratrol and the mediating role of resveratrol targeting protein NQO2 in androgen receptor-positive, hormone-non-responsive CWR22Rv1 cells.. Anticancer Res.

[5] Csiszar A, Labinsky N, Olson S, Pinto JT, Gupte S, Wu JM (2009). Resveratrol prevents monocrotaline-induced pulmonary hypertension in rats.. Hypertension.

[6] Müller C, Ullmann K, Wilkens A, Winterhalter P, Toyokuni S, Steinberg P (2009). Potent antioxidative activity of Vineatrol30 grapevine-shoot extract.. Biosci Biotechnol Biochem.

[7] Kao CL, Tai LK, Chiou SH, Chen YJ, Lee KH, Chou SJ (2010). Resveratrol promotes osteogenic differentiation and protects against dexamethasone damage in murine induced pluripotent stem cells.. Stem Cells Dev.

[8] Oktar S, Ilhan S, Aksulu HE (2008). Clonidine prevents development of hypertension in N (omega)-nitro-L-arginine-treated rats.. Anadolu Kardiyol Derg.

[9] Vapaatalo H, Mervaala E, Nurminen ML (2000). Role of endothelium and nitric oxide in experimental hypertension.. Physiol Res.

[10] Harrison DG, Gongora MC, Guzik TJ, Widder J (2007). Oxidative stress and hypertension.. J Am Soc Hypertens.

[11] Wilcox CS, Welch WJ (2001). Oxidative stress: cause or consequence of hypertension. Exp Biol Med (Maywood).

[12] Silan C, Uzun O, Ustundag Comunoglu N, Gokcen S, Bedirhan S, Cengiz M (2007). Gentamicin induced nephrotoxicity in rats ameroliorated and healing effects of Resveratrol.. Biol Pharm Bull.

[13] Ozkan OV, Yuzbasioglu MF, Ciralik H, Kurutas EB, Yonden Z, Aydin M (2009). Resveratrol, a natural antioxidant, attenuates intestinal ischemia/reperfusion injury in rats.. Tohoku J Exp Med.

[14] Silan C (2008). The effects of chronic resveratrol treatment on vascular responsiveness of streptozotocin-induced diabetic rats.. Biol Pharm Bull.

[15] Sadruddin S, Arora R (2009). Resveratrol: biologic and therapeutic implications.. J Cardiometab Syndr.

[16] Wong RH, Howe PR, Buckly JD, Coates AM, Kunz I, Berry NM (2011). Acute resveratrol supplementation improves flow-mediated dilatation in overweight/obese individuals with mildly elevated blood pressure.. Metab Cardiovasc.

[17] Gojkovic-Bukarica L, Novakovic A, Kanjuh V, Bumbasirevic M, Lesic A, Heinle H (2008). A role of ion channels in the endothelium-independent relaxation of rat mesenteric artery induced by resveratrol.. J Pharmacol Sci.

[18] Zhang HY, Xu CQ, Li HZ, Li BX, Zhang YN, Zhang YQ (2005). Effects of resveratrol on isolated thoracic aorta rings of rats.. Zhongguo Zhong Yao Za Zhi.

[19] Leblais V, Krisa S, Valls J, Courtois A, Adbelouhab D, Villa AM (2008). Relaxation induced by red wine polyphenolic compounds in rat pulmonary arteries: lack of inhibition by NO-synthase inhibitor.. Fundam ClinPharmacol.

[20] Deniz E, Sahna E, Aksulu HE (2006). Nitric oxide synthase inhibition in rats: melatonin reduces blood pressure and ischemia/reperfusion-induced infarct size.. Scand Cardiovasc J.

[21] Nabha L, Garbern JC, Buller CL, Charpie JR (2005). Vascular oxidative stress precedes high blood pressure in spontaneously hypertensive rats.. Clin Exp Hypertens.

[22] Hu L, Zhang Y, Lim PS, Miao Y, Tan C, McKenzie KU (2006). Apocynin but not L-arginine prevents and reverses dexamethasone-induced hypertension in the rat.. Am J Hypertens.

[23] Schnackenberg CG, Welch WJ, Wilcox CS (1998). Normalization of blood pressure and renal vascular resistance in SHR with a membrane-permeable superoxide dismutase mimetic: role of nitric oxide.. Hypertension.

[24] Bhatt SR, Lokhandwala MF, Banday AA (2011). Resveratrol prevents endothelial nitric oxide synthase uncoupling and attenuates development of hypertension in spontaneously hypertensive rats.. Eur J Pharmacol.

[25] Gordish KL, Beierwaltes WH (2014). Resveratrol induces acute endothelium-dependent renal vasodilation mediated through nitric oxide and reactive oxygen species scavenging.. Am J Physiol Renal Physiol.

[26] Turan B, Tuncay E, Vassort G (2012). Resveratrol and diabetic cardiac function: focus on recent in vitro and in vivo studies.. J Bioenerg Biomembr.

[27] Vallance P, Collier J, Moncada S (1989). Effects of endothelium-derived nitric oxide on peripheral arteriolar tone in man.. Lancet.

[28] Granger JP, Alexander BT (2000). Abnormal pressure-natriuresis in hypertension: role of nitric oxide.. Acta Physiol Scand.

[29] Johnson RH, Freeman RH (1992). Pressure natriuresis in rats during blockade of the L-arginine/nitric oxide pathway. Hypertension.

[30] Barrero MJ, Ojeda ML, Díaz Castro J, Nogales F, Murillo ML, Carreras O (2012). The effects of ethanol upon hydric balance and arterial pressure in rats: folic acid as a possible hypotensor.. Life Sci.

[31] Tolins JP, Shultz PJ (1994). Endogenous nitric oxide synthesis determines sensitivity to the pressor effect of salt.. Kidney Int.

[32] Griffin K, Polichnowski A, Licea-Vargas H, Picken M, Long J, Williamson G (2012). Large BP-dependent and -independent differences in susceptibility to nephropathy after nitric oxide inhibition in Sprague- Dawley rats from two major suppliers.. Am Jphysiol Renal Physiol.

